# Retraction: Experimental infection of mice with avian paramyxovirus serotypes 1 to 9

**DOI:** 10.1371/journal.pone.0244048

**Published:** 2020-12-14

**Authors:** The PLOS ONE Editors

After this article [1] was published, concerns were raised about results reported in Figs 1, 2, and 3.

Specifically:

- Concerns were raised about animal ethics considerations for the *in vivo* infection experiments in light of the weight loss reported in Fig 1. In post-publication discussions, the authors stated that animal health was monitored twice per day during the study and that humane endpoints were applied. However, according to the information provided post-publication, the humane endpoint criteria specified that animals should have been euthanized when weight loss exceeded 15%. Fig 1 reports that animals in groups 1, 2, 6, and 9 met or exceeded this limit by day 2 post-infection. In response to this issue, the corresponding author commented that the animal experiments were performed according to the IACUC protocol and that all scores were submitted to the campus veterinarian.
- When levels are adjusted, we did not detect verifiable image content in Fig 2A, 2F, 3b(A), or 3b(F). The authors commented that these control samples did not react with the antibodies and no signal was detected. They provided raw image data which for other panels in the figures aligned with the published results. However, even with background levels and/or brightness adjusted, we did not detect any autofluorescence or background signal on the raw images provided for the panels of concern. Also, the images provided for Fig 3b(A) and Fig 3b(F) each include a white speck that appears the same between the two image files.
- Concerns were raised about whether the experiments reported in Figs 2 and 3 included adequate controls. In post-publication discussions, the authors clarified that for the experiments reported in Figs 2 and 3, they performed only one mock-infected control experiment and used it for a control experiment using only one APMV N-specific antibody. I.e., they did not conduct mock-infection control experiments for every APMV (APMV-1 to APMV-9). Furthermore, they did not report ‘no primary antibody’ or preimmune serum control experiments to demonstrate the sensitivity and/or specificity of the APMV-specific antibodies in immunohistochemistry experiments. Western blot experiments to evaluate the hyperimmune sera were described in the Methods section but these data were not shown. In addition, no controls were reported in the article to demonstrate the efficacy of the viral infections.

The authors offered replication data for Figs 2 and 3 that included additional controls. However, given the nature of the above concerns, we do not consider that replication data fully resolve the issues.

In light of the above issues, the *PLOS ONE* Editors retract this article.

SKS did not agree with retraction. SKK disagreed with retraction and stands by the article’s findings. PLC disagreed with retraction, stands by the article’s findings, and apologized for the issues with the published article. KS andSX either could not be reached or did not respond directly.
